# What could be the fate of secondary contact zones between closely related plant species?

**DOI:** 10.1590/1678-4685-GMB-2019-0271

**Published:** 2020-06-03

**Authors:** Carolina K. Schnitzler, Caroline Turchetto, Marcelo C. Teixeira, Loreta B. Freitas

**Affiliations:** 1 Universidade Federal do Rio Grande do Sul, Departamento de Genética, Laboratório de Evolução Molecular, Porto Alegre, RS, Brazil

**Keywords:** Hybrid zones, gene exchange, introgression, hybridization, Petunia

## Abstract

Interspecific hybridization has been fundamental in plant evolution. Nevertheless, the fate of hybrid zones throughout the generations remains poorly addressed. We analyzed a pair of recently diverged, interfertile, and sympatric *Petunia* species to ask what fate the interspecific hybrid population has met over time. We analyzed the genetic diversity in two generations from two contact sites and evaluated the effect of introgression. To do this, we collected all adult plants from the contact zones, including canonicals and intermediary colored individuals, and compared them with purebred representatives of both species based on seven highly informative microsatellite loci. We compared the genetic diversity observed in the contact zones with what is seen in isolated populations of each species, considering two generations of these annual species. Our results have confirmed the genetic differentiation between the species and the hybrid origin of the majority of the intermediary colored individuals. We also observed a differentiation related to genetic variability and inbreeding levels among the populations. Over time, there were no significant differences per site related to genetic diversity or phenotype composition. We found two stable populations kept by high inbreeding and backcross rates that influence the genetic diversity of their parental species through introgression.

## Introduction

The dispersal of organisms and their gametes leads to the spread of genetic variants. The exchange of genetic variants between diverged lineages is referred to as hybridization, and the geographic areas where hybridization is localized are hybrid zones (Gompert and Buerke, 2016).

Interspecific hybridization has played an essential role in plant evolution, and gene exchange between divergent species can result in new phenotypic or genetic diversity, adaptive variation, and, in some cases, contribute to speciation (Goulet *et al.*, 2017). This process is more likely to happen between closely related species (Abbott *et al.*, 2013; but also see Worth *et al.*, 2016). Hybrid offspring production can promote gene exchange between parental species and, if the hybrids are fertile and can backcross with the parents, this may result in introgression that can lead to genetic swamping if gene exchange is excessive (Todesco *et al.*, 2016) or introduce new, possibly adaptive, genetic combinations (Ellstrand *et al.*, 2013). On the other hand, hybridization between divergent species can favor reproductive isolation by increasing divergence through a process called reinforcement (Hopkins, 2013) due to the cost of low fertility or viability of interspecific hybrids.

Nevertheless, regardless of the importance of interspecific hybridization and the fact that it is frequently studied, the fate of hybrid zones across generations remains poorly addressed. Some controlled experiments have revealed that hybrids can evolve adaptive traits faster than non hybrid under the same conditions, despite the lower initial fines of hybrids (Mitchell *et al.*, 2019), while others demonstrated that a single hybridization event can impact the long-term hybrid survivor (Johansen-Morris and Latta, 2006). Aiming to contribute to the discussion on hybrid zone consequences, we used a pair of recently diverged, interfertile, and sympatric species of annual wildflowers in the genus *Petunia* to ask what fate the interspecific hybrid population meets over time.

The genus *Petunia* contains 14 species that are narrowly distributed in southern South America (Stehmann *et al.*, 2009). The species delimitation can sometimes be problematic (Segatto *et al.*, 2017), but marked morphological differences (Reck-Kortmann *et al.*, 2014) and divergent evolutionary histories (Fregonezi *et al.*, 2013) divide the species into two main clades. Premating reproductive barriers between *Petunia* species appear to be weak (Watanabe *et al.*, 2001), at least in controlled crossings (Gerats and Vandenbussche, 2005). Several morphological traits are shared between species, and a few studies have characterized the genetic diversity of the species with overlapping distribution areas (Lorenz-Lemke *et al.*, 2006; Segatto *et al.*, 2014, 2017), which probably makes difficult to identify interspecific hybrids through molecular markers.

**Figure 1:**
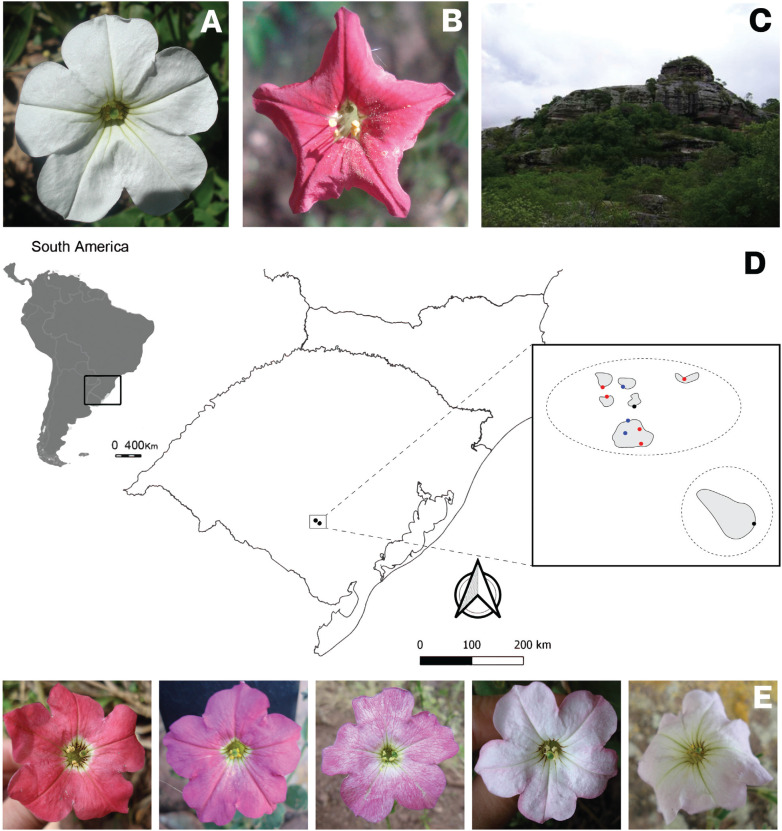
Sampling geographical distribution and morphological characterization. A. *P. axillaris* canonical flower frontal view; B. *P. exserta* canonical flower frontal view; C. CO2 tower; Geographical location and distribution of *P. axillaris* (blue dots), *P. exserta* (red dots), and contact zones (black dots) in Serra do Sudeste, Brazil; E. Intermediary colored flowers in frontal view, from the left to right, class A to E, respectively.

The species *P. axillaris* (Figure 1A) and *P. exserta* (Figure 1B) are sympatric in part of the *P. axillaris* distribution (Lorenz-Lemke *et al.*, 2006; Segatto *et al.*, 2014; Turchetto *et al.*, 2014). They are interfertile sister species (Turchetto *et al.*, 2019b) of annual herbs (Reck-Kortmann *et al.*, 2014), with *P. axillaris* widely distributed in the Pampas in southern Brazil, Uruguay, and Argentina and meeting *P. exserta* in the eastern part of its distribution in Serra do Sudeste (Brazil), where this last species is endemic. Sandstone towers with 300–500 m in elevation interspacing mosaics of open fields in middle of the Pampas biome characterize the Serra do Sudeste region (Figure 1C).

*Petunia axillaris* and *P. exserta* display some contrasting morphological traits, especially concerning their floral shape (Teixeira *et al.*, 2020), and each species occupies different microenvironments. Plants of *P. axillaris* are white, are strongly fragrant at dusk, have hawkmoth-pollinated flowers (Ando *et al.*, 1995; Venail *et al.*, 2010; Klahre *et al.*, 2011) and are found in open and sunny patches on top of or on tower faces. *P. exserta* is bird-pollinated (Lorenz-Lemke *et al.*, 2006) and individuals display bright and intensely red corollas and non-fragrant flowers with exserted stamens, and they grow inside rock cavities (shelters) totally protected from direct sunlight and rain.

Both species blossom in the same period from September to early December and have yellow pollen and long corolla tubes (Stehmann *et al.*, 2009). Along the ca. 3,000 km^2^ in Serra do Sudeste, several populations of each species occupy towers (Turchetto *et al.*, 2014) and shelters (Segatto *et al.*, 2014), forming patches of one to fewer than 50 individuals. In two of these sites (Figure 1D; Table S1), individuals with the typical morphology of *P. axillaris* have been found outside shelters, whereas plants with typical *P. exserta* morphology are found inside shelters together with a variable number of individuals with intermediary corolla color (Figure 1E). The presence of intermediary colored individuals, recorded for the first time in 2002 (Lorenz-Lemke *et al.*, 2006), has been attributed to an intricate pattern of interspecific hybridization, introgression, and ancestral polymorphism sharing in the contact zones (Lorenz-Lemke *et al.*, 2006; Segatto *et al.*, 2014; Turchetto *et al.*, 2015b, 2019a).

Herein, we analyzed the genetic diversity in two generations of individuals from two contact sites and evaluated the effect of introgression on these two *Petunia* species. To do this, we collected all adult plants from the contact zones, including canonicals and intermediary colored individuals, and compared them with purebred representatives of both species based on seven polymorphic microsatellite loci. As intermediary colored individuals were recorded for the first time in 2002 (Lorenz-Lemke *et al.*, 2006) and since than have been found every year at the same sites, we hypothesized that the intermediary individuals are effectively interspecific hybrids, and these populations are stable over time.

## Material and Methods

### Sampling and identification

We collected young leaves of all adult individuals in two flowering seasons (2011 and 2015) from the two known contact zones between *P. axillaris* and *P. exserta*, called CO1 and CO2, totaling 89 individuals. The sampling also included pure stands of *P. axillaris* and *P. exserta* collected in 2011 as reference populations representing the genetic diversity of typical morphologies. For *P. axillaris*, we collected a total of 43 individuals from three sites and for *P. exserta*, 51 individuals from five collection localities (Figure 1D; Table S1), hereafter referred to as isolated sites PaIS1 to PaIS3 and PeIS1 to PeIS5, respectively. We considered these to be isolated sites because they are localities where only one species can be found, and all individuals display all morphological traits that characterize each species based on the description by Stehmann *et al.* (2009).

In the contact zones, the individuals were classified based on their spatial position in relation to shelters (inside or outside) and their flower color as visually inspected against a Red-Green-Blue chart (Table S2): inside cavities, *P. exserta* - red-colored individuals or hybrid classes A to E - intermediary colored flowers; outside cavities, *P. axillaris* - white-flowered plants (Figure 1A,B,E). All individuals were classified from digital pictures taken in nature with a same color scale and at the same conditions.

### DNA extraction and microsatellite genotyping

DNA extraction followed a CTAB-based method (Roy *et al.*, 1992), and we screened a set of seven microsatellites previously described for *P. hybrida* (Bossolini *et al.*, 2011) that are considered informative to identify hybrids between *Petunia* species and are scattered throughout four of seven *Petunia* chromosomes (Turchetto *et al.*, 2015b, Turchetto 2019a). Characteristics of each microsatellite loci are described in Table S3. A three-primer system was employed to label the PCR products fluorescently: forward primers with an M13 tail, an unlabeled reverse primer, and an M13 labeled with fluorescent dyes (FAM, NED, PET or VIC). Microsatellite loci were amplified following the protocol of Turchetto *et al.* (2015b); PCR products were multiplexed and thus denatured and size-fractionated using capillary electrophoresis on an ABI 3100 genetic analyzer (Thermo Fisher Scientific Co., Waltham, USA) with a LIZ (500) molecular size standard (Thermo Fisher Scientific Co.). The Genemarker 1.97 software (Softgenetics LLC, State College, USA) was used to determine the alleles. Additionally, all alleles were visually verified and scored. All individuals exhibited a maximum of two alleles per locus, as expected for diploid species, whose sizes were compatible with repetitions in each locus. All samples were genotyped at the same time and protocols, despite some individuals have been included in other previously published analyses.

### Statistical analyses

We used Micro-Checker software (http://www.microchecker.hull.ac.uk/) to estimate genotyping errors due to stutter bands, allele dropout, and null alleles. Basic frequency statistics [number of alleles, allele richness, number of private alleles, and Nei's unbiased gene diversity (Nei, 1987)] and inbreeding coefficients (*F*_IS_; Weir and Cockerham, 1984) were calculated with FSTAT (Goudet, 1995). As the Wahlund effect can affect the results, we analyzed the sample dividing individuals in three groups: *P. axillaris* (all individuals with white corollas), *P. exserta* (all individuals displaying red corollas), and hybrids (individuals with intermediary colored corollas). As we did not observe the Wahlund effect, we run all analyses considering collection sites as populations (PaIS 1-3, PeIS 1-5, CO1 2011/2015, and CO2 2011/2015). We estimated the observed and expected heterozygosity under Hardy-Weinberg equilibrium after Bonferroni correction and analysis of molecular variance (AMOVA; Excoffier *et al.*, 1992) with 10 000 permutations using Arlequin 3.5 (Excoffier and Licher, 2010). We used Student's *t*-test to compare fluctuations in the number of individuals per generation and collection site considering the seven color classes (red, white, and intermediary A to E) and a Kruskal-Wallis test to compare the diversity indices among populations using the IBM SPSS Statistics 24.0 package (IBM Corp., Armonk, USA).

Using *F*_IS_, we calculated the apparent outcrossing rate (*ta*) for each contact zone in each generation through *ta* = (1 - *F*_IS_)/(1 + *F*_IS_), assuming that predominantly inbreeding populations have ta ≤ 0.2, populations with mixed mating systems show 0.2 <ta ≤ 0.8, and predominantly outcrossing populations have ta >0.8 (Goodwillie *et al.*, 2005).

We generated F_1_, F_2_, and backcross-derived genotypes from isolated sites-based microsatellite profiles of *P. axillaris* and *P. exserta* using Hybridlab 1.0 (Nielsen *et al.*, 2006) to compare polymorphisms between these expected hybrids and the observed hybrids at CO1 and CO2 in both generations. Comparisons were obtained through a Bayesian analysis performed in Structure 2.3.2 software (Pritchard *et al.*, 2000) and carried out under the admixture model assuming independent allele frequencies, using a burn-in period of 250 000, run length of 1 000 000, and ten replicates per K. We used K = 2 corresponding to the two species assuming that both equally contributed to the gene pool of the individuals from the contact zones. Isolated populations of each species were used as reference samples of purebred individuals. We considered as *P. axillaris* the individuals that had a threshold *q* ≤ 0.20 and as *P. exserta* those with *q* ≥ 0.80. Individuals with a threshold 0.20 < *q* <0.80 were classified as hybrids (Burgarella *et al.*, 2009). Subsequently, we assigned a genetic threshold to a spatial distribution relative to the shelter's aperture and visual classification of flower color.

We also performed a Bayesian analysis using NewHybrids 1.1 (Anderson and Thompson, 2002) to assign individuals into six distinct genotype classes (pure *P. axillaris*, pure *P. exserta*, F_1_ hybrid, F_2_ hybrid, F_1_ backcross to pure *P. axillaris*, and F_1_ backcross to pure *P. exserta*). We ran two independent analyses using Jeffrey's priors with uniform priors that included 100,000 steps as burn-in followed by 1,000,000 MCMC interactions to assure the convergence of chains and homogeneity across runs. Analyses were performed without previous information on population or taxonomic identity (Vähä and Primmer, 2006). The posterior probability (PP) of categorical membership for each individual was computed using a Bayesian approach. We used PP ≤ 0.50 to assign individuals to a genotypic class (Murphy *et al.*, 2019). After that, we assigned the genotypic class to the genetic threshold obtained with Structure, spatial distribution relative to the shelter's aperture, and visual classification of flower color.

## Results

### Morphological classification

All collected adult individuals were classified in the field based on the corolla color and continuously numbered (Table 1; Table S2). As expected, from all isolated sites of *P. axillaris*, the individuals were found outside shelter and displayed pure white corollas. Similarly, from isolated sites of *P. exserta*, individuals were uniformly red-colored, and all of them were found inside shelter. Pairwise fluctuations in the number of individuals per site and color class were compared through Student's *t*-test (Table S4), and the number of *P. axillaris* individuals was different between the contact zones and over time, whereas the *P. exserta* number was constant between generations in CO1. Among intermediary colored individuals, only those in class C were constant in number over time and across collection sites. Considering all intermediary classes (A to E) as one unit, we observed significant fluctuation in numbers of individuals in all comparisons.

**Table 1:** Morphological characterization of individuals from the contact zones per generation

		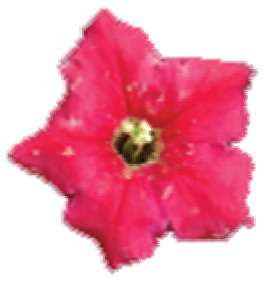 *P. exserta*	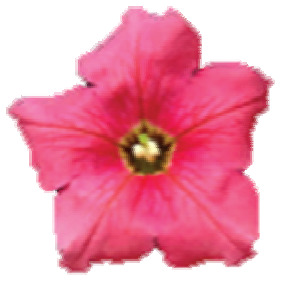 Class A	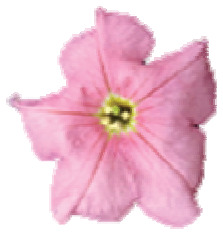 Class B	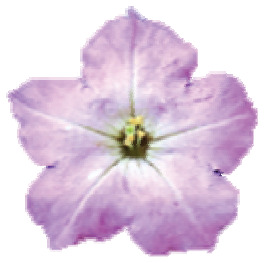 Class C	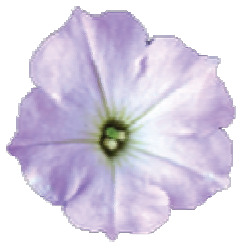 Class D	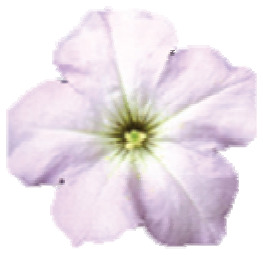 Class E	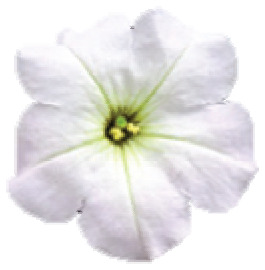 *P. axillaris*
2011	CO1	3	5	0	1	1	3	12
	CO2	12	3	1	0	0	1	1
2015	CO1	1	1	0	3	0	0	16
	CO2	7	5	1	1	2	0	8

CO1 and CO2 - contact zones; Class A to E - intermediary colored individuals based on RBG chart

### Mating system and genetic diversity

All pairs of loci were in linkage equilibrium (P < 0.001, Bonferroni's adjusted value for a nominal level of 5%), and all loci and collection sites were polymorphic (Table S5). The frequency of null alleles was low (< 0.5%) across all loci and we did not found genotyping errors. Considering the total diversity per collection site per year (Table 2), we observed that all sites had a deficit of heterozygotes and high and significant *F*_IS_ values, suggesting inbreeding. The highest inbreeding value was observed in isolated sites of *P. exserta*, followed by CO2 during the 2015 flowering season. All populations, except *P. exserta* from isolated sites, showed a predominant mixed mating system based on apparent crossing rate (*ta*). Allele richness and genetic diversity did not differ between sites between years, whereas observed heterozygosity and inbreeding coefficient varied between sites and years. The highest *F*_IS_ was observed in PeIS, whereas this value significantly decreased in CO1 over the years with the opposite situation in CO2 (Table 2).

**Table 2:** Genetic diversity and predominant mating system per collection site per year considering seven microsatellite loci

	PaIS	PeIS	CO1		CO2	
			2011	2015	2011	2015
N	50	35	44	43	36	34
E	8	3	3	4	1	3
H_O_	**0.412**	**0.123**	**0.299**	**0.286**	**0.373**	**0.267**
H_E_	0.666	0.486	0.698	0.577	0.734	0.603
R	6.081	4.418	5.910	6.117	5.134	4.720
GD	0.665	0.490	0.706	0.743	0.568	0.611
*F*_IS_	0.387	0.762	0.577	0.496	0.497	0.564
ta	0.442	0.135	0.268	0.337	0.336	0.279

PaIS - *P. axillaris* isolated sites; PeIS - *P. exserta* isolated sites; CO – contact sites N – number of alleles; H_O_ - observed heterozygosity; H_E_ - expected heterozygosity; E – number of private alleles; R – allele richness; GD – Nei's unbiased gene diversity; *F*_IS_ – inbreeding coefficient; ta - apparent crossing rate according Goodwillie *et al.* (2005). Values in bold – HWE deviation after Bonferroni's correction (P <0.05).

### Genetic identity

**Figure 2:**
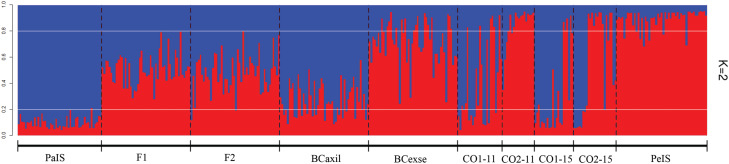
Structure bar plot under admixture coefficients model based on seven microsatellite loci considering individuals from allopatric populations of *Petunia exserta* (PeIS) and *P. axillaris* (PaIS), intermediary colored individuals (CO1 and CO2 in two generations, 2011 and 2015), and F_1_, F_2_, and backcrosses derived genotypes obtained using Hybridlab. Each bar represents individuals and black-dotted vertical lines separate collection sites and generations; different colors correspond to genetic components (K = 2) and individuals' membership. Horizontal white lines indicate thresholds q = 0.2 and q = 0.8

Individuals from isolated sites of *P. axillaris* were purebred according to our criteria in Structure analysis (Figure 2), whereas a few individuals of *P. exserta* had a *P. axillaris* component despite always having *q* < 0.7.

The simulated genotypes with Hybridlab, independent of whether it was from the F_1_ or F_2_ generation, showed a threshold range of 0.20 < *q* < 0.80 (hybrids), whereas when the backcrosses were considered, we observed purebred individuals of each species and hybrids. Individuals from contact zones across generations displayed a varied pattern of thresholds, including values from those of each species to those as expected from F_1_, F_2_, and backcrosses and the observed threshold was not always concordant with the visual classification of corolla color. Considering both contact zones and the two generations, ca. 86% of white-flowered individuals had the *P. axillaris* genetic component, whereas ca. 83% of red-colored flowers were assigned to the *P. exserta* genetic group. Intermediary colored individuals were preferentially (71%) assigned as *P. exserta* (Table S2).

**Figure 3:**
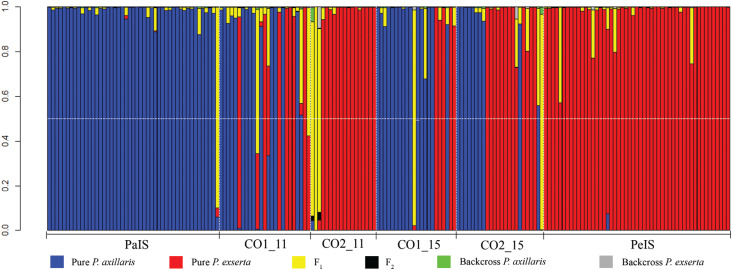
Posterior probabilities (PP) for all analyzed plants using NewHybrids assigned to six classes following the color legend for pure parental species (*P. axillaris* or *P. exserta*), F_1_, F_2_, backcrosses (BC) with *P. axillaris,* and backcrosses with *P. exserta*. Vertical black dotted lines separate collection sites and generations, and the horizontal white dotted line indicates PP = 0.5.

Based on the NewHybrids analysis (Figure 3), we only observed purebred individuals of each species or F_2_ hybrids, independent of collection site and reproductive season, and considered PP ≥ 0.50 to assign individuals to each genotypic class. All individuals except one collected in isolated sites of *P. axillaris* were classified as purebred *P. axillaris* individuals. That F_2_ individual displayed a white corolla color, was found outside shelter, and showed a threshold *q* ≤ 0.20 in Structure analysis. All individuals from PeIS were classified as purebred *P. exserta* individuals. The composition of the contact zones was different with purebred *P. axillaris* individuals found more frequently in CO1 in both generations, whereas in CO2, we observed a higher number of purebred *P. exserta* individuals. Only one individual remained unclassified based on PP, and it showed a hybrid threshold in Structure and corolla color class A (individual ID 14a from CO1). Concordance among phenotypes (corolla color), Structure, and NewHybrids classifications was observed more frequently (∼60% of individuals) than discrepancies. Corolla color and Structure classifications were concordant for 70% of individuals, whereas color class and NewHybrids agreed in 65% of cases. Structure and NewHybrids classifications were concordant 83% of the time (Table S2).

## Discussion

Herein we described the genetic diversity at two sites where interspecific crosses between *P. axillaris* and *P. exserta* occur, considering two generations of crosses that resulted in the production of individuals with atypical morphology and intermediary corolla color between the parental species. We based the analysis on highly variable and informative microsatellite loci (Turchetto *et al.*, 2015b, 2019a), and compared the genetic diversity observed in contact zones with that seen in isolated populations composed only of individuals with the canonical morphology of each species, considering two generations separated by five reproductive seasons of these two annual and herbaceous species. Our results confirmed the genetic differentiation between the species and the hybrid origin of the majority of intermediary colored individuals. We also observed a differentiation related to genetic variability and inbreeding levels among the populations. Over time, there were no significant differences per site related to genetic diversity and all the same phenotypes were found (canonical morphology of each species and intermediary colored individuals).

The role that hybridization plays in different taxa or populations is variable (Taylor and Larson, 2019). What happens after hybridization is dependent on reproductive barriers between hybrids and their parental strains and, at least for animal-pollinated species, depends on whether hybrid self-fertilization increases (Jordan *et al.*, 2018) and stable populations are established. Our results have indicated that, in the contact zones, the populations are stable in regards to the number of individuals and kinds of phenotypes found over time and that introgression is frequent in both species groups as canonical phenotypes include individuals with mixed genetic components (see Structure results) or those resulting from backcrosses (see NewHybrids results).

Introgressive hybridization can be beneficial or detrimental to biodiversity (Ellstrand, 2001). Here, we described intermediary colored individuals, showing an undoubtedly hybrid genetic constitution, growing inside shelters and some others that were found outside shelters. As genetic diversity and morphological polymorphisms are kept throughout different generations, we suggest that hybridization and bidirectional introgression involving these *Petunia* species are beneficial to them and increase their variability.

A recent study conducted in these contact areas on morphological differences among hybrids and canonical individuals, including five floral traits, showed that intermediary colored individuals from CO1 are also morphologically intermediary between canonical individuals of *P. axillaris* and *P. exserta*, whereas putative hybrids from CO2 are more similar to *P. exserta* (Turchetto *et al.*, 2019a). These results are in agreement with our Structure results and could be explained based on mating dynamics observed throughout the generations in each contact zone (see NewHybrids analysis).

Several authors have pointed out that inbreeding could be a strategy to maintain the species' limits and avoid introgression between different species (Lowe and Abbott, 2004; Martin and Willis, 2007). In particular, self-fertilization has been considered to be a highly effective way to establish species or lineages during the colonization of new environments (Kamran-Disfani and Agrawal, 2014). Concerning these *Petunia* species in the Serra do Sudeste region, we can observe high inbreeding levels and a shift in the mating system (from self-incompatible to a mixed system), at least for *P. axillaris* (Turchetto *et al.*, 2015a). *P. exserta* has been described as a self-compatible plant (Stehmann *et al.*, 2009).

Different studies have assumed that species boundaries in *Petunia* are maintained primarily by the strong specificity of their pollinators (Stuurman *et al.*, 2004; Sheehan *et al.*, 2016). Although *P. axillaris* is primarily pollinated by hawkmoths, some bees and hummingbirds have been observed visiting its flowers (Lorenz-Lemke *et al.*, 2006; Gübitz *et al.*, 2009). *P. exserta* flowers display a set of traits usually seen in bird-pollinated species but share with *P. axillaris* some scent compounds that are attractive to bees (Rodrigues *et al.*, 2018).

In addition, selfing can favor pioneer species (Cheptou, 2019) or those that live under adverse ecological conditions (Levin, 2012). The environment where *P. exserta* and the majority of hybrids grow is considered inhospitable to other *Petunia* species (Stehmann *et al.*, 2009) and, in the contact zones, the observed high inbreeding frequency likely has guaranteed the maintenance of individuals and these populations' stability, with inbreeding and a mixed mating system predominating among populations (see *ta* values). Mixed mating systems and high levels of inbreeding have also explained, at least in part, the genetic diversity (Rodrigues *et al.*, 2019) and maintenance of species limits (Turchetto *et al.*, 2015a) in other *Petunia* species when these taxa occur in restrictive environments or on the borders of their geographical distribution.

The pronounced structure observed mainly in contact zones and *P. exserta* populations can be associated with the exclusive occurrence of this species and intermediary colored individuals inside shelters that are not physically connected (Segatto *et al.*, 2014). *P. exserta* has low potential for seed dispersal (van der Pijl, 1982) and its populations are usually small and substantially isolated (Lorenz-Lemke *et al.*, 2006), which may favor genetic drift within each local population. Reproductive isolation in combination with relatively strong natural selection or genetic drift may promote rapid speciation (Escudero *et al.*, 2019), which has been broadly documented for *Petunia* species (Ando *et al.*, 2005; Kulcheski *et al.*, 2006; Fregonezi *et al.*, 2013). The high *F*_IS_ values found in these populations also reinforce the hypothesis of evolution in local populations under the influence of genetic drift and inbreeding.

Here, as in other species (i.e., Mota *et al.*, 2019), *P. exserta* is kept as a unique evolutionary unit, independently from *P. axillaris*, but contrarily to those Bromeliads, the gene exchange is not low between *Petunia* species in Serra do Sudeste (see Turchetto *et al.*, 2019b). *P. exserta* can be considered narrowly endemic compared to *P. axillaris* and, in general, has lower diversity indices than its sister species even accounting only sympatric populations (Lorenz-Lemke *et al.*, 2006; Segatto *et al.*, 2014). Extensive and continuous gene exchanging could impact *P. exserta* survivor, especially in light of an easy potencial shift in pollinators. As previously showed (Hermann *et al.*, 2013), all differential traits between *P. exserta* and *P. axillaris* involved into the pollinator attraction are linked in a very short chromosome segment and hybridization could promote recombination among them.

Our analyses confirmed the hybrid status of individuals from contact zones that are also intermediary in morphology based on their corolla color. The patterns of hybridization observed here were not uniform in the two contact zones because they were not limited to the hybridization first generation (see NewHybrids results), indicating long-term hybridization, back-crosses, and historic introgression between *P. axillaris* and *P. exserta.* The NewHybrids analysis can underestimate backcrosses when parental species have recently diverged, classifying the individuals as purebreds of one or another parent (Vähä and Primmer, 2006).

Some individuals from allopatric populations presented signals of hybridization, which can be interpreted as a consequence of historical gene exchange, retention of ancestral polymorphisms or even a small degree of long-distance gene flow through the pollen. Ancestral polymorphism sharing between *P. axillaris* and *P. exserta* has been observed through plastid haplotype sharing among individuals of both species collected in the same towers, despite finding them in different patches inside or outside shelters (Lorenz-Lemke *et al.*, 2006). Evolutionary proximity between these two species has also been widely supported by nuclear markers (Reck-Kortmann *et al.*, 2014; Segatto *et al.*, 2014) and through genomic analysis (Esfeld *et al.*, 2018). At least for *P. axillaris*, pollen flow is restricted to short distances (Turchetto *et al.*, 2015a) along with gene exchange between *P. axillaris* and *P. exserta* (Turchetto *et al.*, 2019b).

Interspecific hybridization in zones of secondary contact after allopatric divergence is frequently observed between closely related species with overlapping distributions (Taylor *et al.*, 2014; Ley and Hardy, 2017), such as *P. axillaris* and *P. exserta* (Lorenz-Lemke *et al.*, 2006). Our results suggest that differences in distribution patterns between *P. axillaris* and *P. exserta* could reflect their preferences for landscape and habitat characteristics, likely as a consequence of distinct evolutionary histories rather than simple ancestral polymorphism sharing as previously thought based on plastid markers (Kulcheski *et al.*, 2006; Lorenz-Lemke *et al.*, 2006) and some nuclear markers (Segatto *et al.*, 2014).

Furthermore, the existence of only two contact zones with a low frequency of hybrids between two *Petunia* species in an area of more than 3,000 km^2^ and dozens of populations of each species occupying towers (Turchetto *et al.*, 2014) and shelters (Segatto *et al.*, 2014), forming patches of one to fewer than 50 individuals, indicate that continuous hybridization is limited.

In sum, we are facing two stable hybrid populations that influence the genetic diversity of their parental species. These sites are mainly maintained by high inbreeding and backcross rates and could initially be formed through pollen exchange between *P. axillaris* and *P. exserta* by secondary or non-specific pollinators.

## Supplementary material

The following online material is available for this article:

Table S1 - Geographical distribution and sampling.

Table S2 - Morphological and molecular characterization of individuals collected in contact zones.

Table S3 - Microsatellites used to characterize the genetic diversity of *Petunia axillaris, P. exserta,* and intermediary colored individuals.

Table S4 - Pairwise fluctuation in the number of individuals per site per generation (Student's *t*-test).

Table S5 - Diversity indices per locus per collection site per year.
