# Supplementary material for: What could be the fate of secondary contact zones between closely related plant species?
Source: Genet Mol Biol. 2020 Jun 3;43(2):e20190271. doi: 10.1590/1678-4685-GMB-2019-0271 (PMC7299303; doi:10.1590/1678-4685-GMB-2019-0271)
Supplement: Supplementary file 1 [file 1415-4757-GMB-43-2-e20190271-s1.pdf]

Supplementary Material to: "What could be the fate of secondary contact zones between closely related plant species?"

Table S1 - Geographical distribution and sampling.

Site code	Geographic Coordinates	N	
		2011	2015
PaIS1	30° 50' 20.938"S / 53° 30' 14.914"W	17	
PaIS2	30° 50' 10.886"S / 53° 30' 18.512"W	15	
PaIS3	30° 50' 20.430"S / 53° 30' 13.390"W	15	
PeIS1	30° 50' 20.190"S / 53° 30' 12.318"W	8	
PeIS2	30° 49' 56.000"S / 53° 29' 47.000"W	7	
PeIS3	30° 50' 22.000"S / 53° 30' 12.000"W	12	
PeIS4	30° 50' 12.422"S / 53° 30' 22.484"W	6	
PeIS5	30° 50' 09.000"S / 53° 30' 24.000"W	18	
CO1	30° 53' 48.153"S / 53° 25' 16.080"W	25	22
CO2	30° 50' 13.761"S / 53° 30' 15.036"W	18	24

PaIS – *P. axillaris* isolated sites; PeIS – *P. exserta* isolated sites; CO1 and CO2 – co-occurring sites; N – number of collected individuals in each season.
