# Supplementary material for: What could be the fate of secondary contact zones between closely related plant species?
Source: Genet Mol Biol. 2020 Jun 3;43(2):e20190271. doi: 10.1590/1678-4685-GMB-2019-0271 (PMC7299303; doi:10.1590/1678-4685-GMB-2019-0271)
Supplement: Supplementary file 2 [file 1415-4757-GMB-43-2-e20190271-s2.pdf]

Supplementary Material to: "What could be the fate of secondary contact zones between closely related plant species?"

Table S2 - Morphological and molecular characterization of individuals collected in contact zones.

Site	Season 2011					Season 2015				
	ID	Spatial position	Color	Threshold	NH	ID	Spatial position	Color	Threshold	NH
CO1	1a	out	white	$q \leq 0.20$	*P. axillaris*	1b	out	white	$q \leq 0.20$	*P. axillaris*
	2a	out	white	$q \leq 0.20$	*P. axillaris*	2b	out	white	$q \leq 0.20$	*P. axillaris*
	3a	out	white	$0.20 < q < 0.80$	*P. axillaris*	3b	out	white	$0.20 < q < 0.80$	*P. axillaris*
	4a	out	white	$q \leq 0.20$	*P. axillaris*	4b	out	white	$q \leq 0.20$	*P. axillaris*
	5a	out	white	$0.20 < q < 0.80$	*P. axillaris*	5b	out	white	$q \leq 0.20$	*P. axillaris*
	6a	in	A	$q \geq 0.80$	*P. exserta*	6b	out	white	$q \leq 0.20$	*P. axillaris*
	7a	out	white	$q \leq 0.20$	*P. axillaris*	7b	out	white	$q \leq 0.20$	*P. axillaris*
	8a	in	E	$q \leq 0.20$	*P. axillaris*	8b	out	white	$q \leq 0.20$	*P. axillaris*
	9a	out	white	$q \leq 0.20$	*P. axillaris*	9b	out	white	$q \leq 0.20$	*P. axillaris*
	10a	out	white	$q \leq 0.20$	*P. axillaris*	10b	out	white	$q \leq 0.20$	*P. axillaris*
	11a	in	red	$0.20 < q < 0.80$	F_2	11b	out	white	$0.20 < q < 0.80$	F_2
	12a	in	D	$0.20 < q < 0.80$	*P. axillaris*	12b	out	white	$q \leq 0.20$	*P. axillaris*
	13a	in	A	$q \geq 0.80$	*P. exserta*	13b	out	white	$q \leq 0.20$	*P. axillaris*
	14a	in	A	$0.20 < q < 0.80$	ND	14b	out	white	$0.20 < q < 0.80$	*P. axillaris*
	15a	out	white	$q \leq 0.20$	*P. axillaris*	15b	out	white	$q \leq 0.20$	*P. axillaris*
	16a	out	white	$q \leq 0.20$	*P. axillaris*	16b	out	white	$q \leq 0.20$	*P. axillaris*
	17a	in	E	$0.20 < q < 0.80$	*P. exserta*	17b	in	red	$q \geq 0.80$	*P. exserta*
	18a	out	white	$q \leq 0.20$	*P. axillaris*	18b	in	C	$q \geq 0.80$	*P. exserta*
	19a	in	A	$q \geq 0.80$	*P. exserta*	19b	in	A	$q \geq 0.80$	*P. exserta*

Table S2 – Continuation...

	20a	in	A	$q \geq 0.80$	*P. exserta*	20b	in	C	$0.20 < q < 0.80$	*P. axillaris*
	21a	in	red	$q \geq 0.80$	*P. exserta*	21b	in	A	$q \geq 0.80$	*P. exserta*
	22a	out	white	$q \leq 0.20$	*P. axillaris*	22b	in	C	$0.20 < q < 0.80$	*P. exserta*
	23a	in	C	$0.20 < q < 0.80$	*P. axillaris*					
	24a	in	red	$q \geq 0.80$	*P. exserta*					
	25a	in	E	$0.20 < q < 0.80$	F_2					
	26a	out	white	$0.20 < q < 0.80$	F_2	23b	out	white	$q \leq 0.20$	*P. axillaris*
	27a	in	B	$0.20 < q < 0.80$	F_2	24b	out	white	$q \leq 0.20$	*P. axillaris*
	28a	in	red	$0.20 < q < 0.80$	F_2	25b	out	white	$q \leq 0.20$	*P. axillaris*
	29a	in	E	$0.20 < q < 0.80$	*P. exserta*	26b	out	white	$q \leq 0.20$	*P. axillaris*
	30a	in	red	$q \geq 0.80$	*P. exserta*	27b	out	white	$q \leq 0.20$	*P. axillaris*
	31a	in	red	$0.20 < q < 0.80$	*P. exserta*	28b	out	white	$q \leq 0.20$	*P. axillaris*
	32a	in	red	$q \geq 0.80$	*P. exserta*	29b	out	white	$q \leq 0.20$	*P. axillaris*
	33a	in	red	$q \geq 0.80$	*P. exserta*	30b	out	white	$0.20 < q < 0.80$	*P. axillaris*
	34a	in	A	$q \geq 0.80$	*P. exserta*	31b	in	red	$q \geq 0.80$	*P. exserta*
CO2	35a	in	red	$q \geq 0.80$	*P. exserta*	32b	in	red	$q \geq 0.80$	*P. exserta*
	36a	in	red	$q \geq 0.80$	*P. exserta*	33b	in	red	$q \geq 0.80$	*P. exserta*
	37a	in	red	$q \geq 0.80$	*P. exserta*	34b	in	red	$q \geq 0.80$	*P. exserta*
	38a	in	red	$q \geq 0.80$	*P. exserta*	35b	in	red	$q \geq 0.80$	*P. exserta*
	39a	in	A	$q \geq 0.80$	*P. exserta*	36b	in	red	$q \geq 0.80$	*P. exserta*
	40a	in	red	$q \geq 0.80$	*P. exserta*	37b	in	red	$q \geq 0.80$	*P. exserta*
	41a	in	red	$q \geq 0.80$	*P. exserta*	38b	in	A	$q \geq 0.80$	*P. exserta*
	42a	in	red	$q \geq 0.80$	*P. exserta*	39b	in	A	$q \geq 0.80$	*P. exserta*
	43a	in	A	$q \geq 0.80$	*P. exserta*	40b	in	D	$q \leq 0.20$	*P. axillaris*
						41b	in	B	$q \geq 0.80$	*P. exserta*
						42b	in	C	$0.20 < q < 0.80$	*P. exserta*
						43b	in	A	$q \geq 0.80$	*P. exserta*

Table S2 – Continuation...

	44b	in	A	$q \geq 0.80$	*P. exserta*
	45b	in	D	$0.20 < q < 0.80$	*P. axillaris*
	46b	in	A	$0.20 < q < 0.80$	F_2

CO – contact zones 1 and 2, respectively; ID – individual code according season collection (a – 2011; b – 2015); class – classification of each individual based on corolla color: A to E – intermediary corolla color following RGB chart; white – *P. axillaris* typical corolla color; red – *P. exserta* typical corolla color; *q* – genetic component as obtained in STRUCTURE analysis (*P. axillaris* $q \leq 0.20$; *P. exserta* $q \geq 0.80$; hybrids $0.20 < q < 0.80$); NH – genotype class as obtained in NEWHYBRIDS analysis (*P. axillaris* - purebred *P. axillaris*; *P. exserta* - purebred *P. exserta*; F_2 - second generation hybrid); ND – not determined. For more information on intermediary colored individuals see Fig. 1 in main text. out - outside shelters; in - inside shelters.
