# Supplementary material for: What could be the fate of secondary contact zones between closely related plant species?
Source: Genet Mol Biol. 2020 Jun 3;43(2):e20190271. doi: 10.1590/1678-4685-GMB-2019-0271 (PMC7299303; doi:10.1590/1678-4685-GMB-2019-0271)
Supplement: Supplementary file 3 [file 1415-4757-GMB-43-2-e20190271-s3.pdf]

Supplementary Material to: "What could be the fate of secondary contact zones between closely related plant species?"

Table S3 - Microsatellites used to characterize genetic diversity of *Petunia axillaris, P. exserta,* and intermediary colored individuals.

Chromosome	Locus	Primer sequences	Ta (°C)	Repeat
I	PM188	F: CCCAACCATTGGCTACAGCC<br>R: GGACAACACAATACAATCTCTGC	51	$(CTT)_8$
I	PM195	F: GCCTTTCGCCGCTGTCACTG<br>R: GAGCAAATCGTGACCGTTGG	50	$(GAA)_6$
II	PM21	F: CTACCGGTAGGCAGTAGTTGC<br>R: CCTCGACCTTCTTCCTGAC	50.5	$(TAC)_8$
IV	PM8	F: TCTGCAAACTTCAAAGCCAA<br>R: ACATGCCATGCACTTTTGAG	50	$(AAGA)_{10}$
IV	PM173	F: CAGCGCTATCAACAGCAG<br>R: GTGAGAGGCAAGTGATTGG	51	$(GCA)_6$
V	PM167	F: CTCACTAACCAACTTCACC<br>R: CTAAGAAGCTTAAGAGTG	52	$(TTC)_{12}$
V	PM177	F: CCCTTACTCTCTTCTTCACC<br>R: GAACTATGAACCATAGCTCTC	50	$(CA)_{11}$

Ta – Annealing temperature.
