# Supplementary material for: What could be the fate of secondary contact zones between closely related plant species?
Source: Genet Mol Biol. 2020 Jun 3;43(2):e20190271. doi: 10.1590/1678-4685-GMB-2019-0271 (PMC7299303; doi:10.1590/1678-4685-GMB-2019-0271)
Supplement: Supplementary file 4 [file 1415-4757-GMB-43-2-e20190271-s4.pdf]

Supplementary Material to: "What could be the fate of secondary contact zones between closely related plant species?"

Table S4 - Pairwise fluctuation in number of individuals per site per generation (Student's t-test).

	CO1 (2011) vs. CO2 (2011)	CO1 (2015) vs. CO2 (2015)	CO1 (2011) vs. CO1 (2015)	CO2 (2011) vs. CO2 (2015)
Pa	$P < 0.05$	$P < 0.05$	$P < 0.05$	$P < 0.05$
Pe	$P < 0.05$	$P < 0.05$	n.s.	$P < 0.05$
A	n.s.	n.s.	$P < 0.05$	n.s.
B	n.s.	$P < 0.05$	n.s.	n.s.
C	n.s.	n.s.	n.s.	n.s.
D	n.s.	n.s	n.s.	$P < 0.05$
E	n.s.	n.s.	$P < 0.05$	n.s.
A to E	$P < 0.05$	$P < 0.05$	$P < 0.05$	$P < 0.05$

n.s. – not significant at $\alpha = 0.05$; Pa – *Petunia axillaris* (white) color; Pe – *Petunia exserta* (red) color; A to E – intermediary color classes; CO – contact zones sampled during two flowering seasons (2011 and 2015). A to E - all intermediary colored individuals per site per generation.
