# Supplementary material for: What could be the fate of secondary contact zones between closely related plant species?
Source: Genet Mol Biol. 2020 Jun 3;43(2):e20190271. doi: 10.1590/1678-4685-GMB-2019-0271 (PMC7299303; doi:10.1590/1678-4685-GMB-2019-0271)
Supplement: Supplementary file 5 [file 1415-4757-GMB-43-2-e20190271-s5.pdf]

Supplementary Material to: "What could be the fate of secondary contact zones between closely related plant species?"

Table S5 - Diversity indices per locus per collection site per year.

Site	Locus	SR	P	N	GD	R	H_O	H_E	F_{IS}
**PaIS**	PM177	202-256	3	18	0.768	13.131	0.638	0.766	0.169
	PM188	124-145	1	8	0.836	7.465	0.468*	0.832	0.440
	PM195	199-214	0	4	0.573	3.729	0.227*	0.596	0.604
	PM21	125-131	0	3	0.605	2.999	0.425*	0.603	0.296
	PM8	163-183	0	4	0.652	3.996	0.383*	0.649	0.413
	PM167	291-306	1	6	0.706	5.442	0.447*	0.703	0.367
	PM173	154-178	3	7	0.517	5.628	0.298*	0.515	0.424
	**Total**		8	50	0.665	6.081	0.412*	0.666	0.387
**PeIS**	PM177	212-256	1	11	0.873	9.746	0.176*	0.866	0.798
	PM188	124-139	0	5	0.321	4.047	0.080*	0.318	0.751
	PM195	175-214	1	4	0.185	3.276	0.039*	0.184	0.789
	PM21	125-137	1	3	0.428	2.412	0.098*	0.425	0.771
	PM8	175-179	0	2	0.269	2.000	0.039*	0.267	0.854
	PM167	294-306	0	4	0.597	3.794	0.157*	0.592	0.737
	PM173	166-196	0	6	0.759	5.652	0.274*	0.754	0.638
	**Total**		3	35	0.490	4.418	0.123*	0.486	0.762
**CO1 2011**	PM177	214-250	1	10	0.845	8.746	0.375*	0.835	0.556
	PM188	121-142	0	6	0.625	5.844	0.120*	0.615	0.808
	PM195	193-214	1	6	0.788	5.837	0.333*	0.778	0.577
	PM21	125-137	0	4	0.465	3.901	0.240*	0.460	0.484
	PM8	163-183	0	4	0.707	3.919	0.458*	0.701	0.351
	PM167	279-303	1	7	0.683	6.542	0.208*	0.673	0.695
	PM173	166-196	0	7	0.833	6.580	0.360*	0.823	0.568
	**Total**		3	44	0.706	5.910	0.299*	0.698	0.577
**CO2 2011**	PM177	212-256	1	9	0.776	9.000	0.294*	0.761	0.621
	PM188	121-136	0	5	0.650	4.997	0.056*	0.633	0.915
	PM195	175-214	0	5	0.369	5.000	0.294	0.367	0.204
	PM21	125-137	0	3	0.448	2.998	0.111*	0.438	0.752
	PM8	163-179	0	3	0.479	3.000	0.333	0.475	0.304
	PM167	285-306	0	6	0.729	5.943	0.500*	0.722	0.314
	PM173	166-196	0	5	0.653	5.000	0.412*	0.645	0.369

Table S5 – Continuation…

Site	Locus	SR	P	N	GD	R	H_O	H_E	F_{IS}
	**Total**		1	36	0.568	5.134	0.373*	0.734	0.497
**CO1 2015**	PM177	212-258	1	11	0.906	11.000	0.524*	0.897	0.422
	PM188	124-142	0	6	0.738	6.000	0.571*	0.734	0.226
	PM195	193-214	0	5	0.712	4.955	0.091*	0.698	0.872
	PM21	125-131	0	3	0.557	3.000	0.381	0.553	0.316
	PM8	163-183	0	4	0.723	4.000	0.273*	0.712	0.623
	PM167	279-309	3	9	0.779	8.862	0.318*	0.768	0.592
	PM173	166-196	0	5	0.787	5.000	0.454*	0.779	0.422
	**Total**		4	43	0.743	6.117	0.286*	0.577	0.496
**CO2 2015**	PM177	212-256	0	10	0.825	9.565	0.391*	0.815	0.526
	PM188	124-142	0	5	0.562	4.864	0.318*	0.556	0.434
	PM195	190-214	1	5	0.676	4.985	0.208*	0.666	0.692
	PM21	119-128	1	3	0.511	2.875	0.208*	0.504	0.592
	PM8	175-179	0	2	0.504	2.000	0.167*	0.496	0.669
	PM167	291-306	0	4	0.587	4.000	0.286*	0.579	0.513
	PM173	166-199	1	5	0.615	4.750	0.292*	0.608	0.526
	**Total**		3	34	0.611	4.720	0.267*	0.603	0.564

SR – allele size range (in base pairs); E – number of private alleles; N – number of alleles; GD – Nei's unbiased gene diversity; R – allele richness; H_O – observed heterozygosity; H_E – expected heterozygosity under Hardy-Weinberg equilibrium; F_{IS} – inbreeding coefficient; * significant deviation from Hardy-Weinberg equilibrium after Bonferroni's correction ($P < 0.05$).
